# Hybrid Physical Gels from Polymers and Self-Assembled Systems: A Novel Path for Making Functional Materials

**DOI:** 10.3390/gels4020035

**Published:** 2018-04-16

**Authors:** Jean-Michel Guenet

**Affiliations:** Institut Charles Sadron, CNRS-Université de Strasbourg, 23 rue du Loess, BP 84047, 67034 Strasbourg Cedex2, France; jean-michel.guenet@ics-cnrs.unistra.fr; Tel.: +33-388-414-145

**Keywords:** polymer thermoreversible gels, self-assembled systems, functional materials

## Abstract

In recent years, the synthesis of novel organic molecules that spontaneously self-assemble into a large variety of molecular architecture, particularly the formation of organogels, has yielded new opportunities in the preparation of functional materials. Here, we present an original preparation path of such materials through the fabrication of hybrid gels of these molecules with covalent polymers. Three types of systems are described: (i) intermingled gels where a polymer gel and an organogel pervade one another; (ii) encapsulation of self-assembled filaments in polymer fibrils, which provides a system with unusual magnetic properties; (iii) the reverse situation in which self-assembled nanotubes sheathe polymer fibrils. Two covalent polymers are considered: a neutral polymer, specifically stereoregular polystyrene (isotactic or syndiotactic), and a semi-conducting polymer, P3BT. In the latter case, semi-conducting nanowires are obtained.

## 1. Introduction

The past two decades have witnessed the thriving of several new organic molecules capable of self-assembling that, once in solution, subsequently generate a large variety of molecular architectures such as filaments, fibrils, and platelets [1,2,3,4,5,6]. In many cases, the resulting macroscopic state consists of a gel-like or a paste-like substance. Interestingly, some of these molecules also possess functional properties [2] (e.g., magnetic, opto-electronic, electronic, etc that can open new horizons for developing functional materials in replacement for and/or in addition to current natural compounds (for instance, the replacement of rare earths would be a tremendous achievement). Yet, in many cases, these systems cannot be processed into functional materials for various reasons including limited available quantities because of their complex syntheses, their cost, and/or poor mechanical properties due to the use of low concentrations.

As a rule, a functional material is rarely obtained from a pure compound. The best example is provided by metallic alloys whose properties depend upon the subtle mixture of different metals. In the realm of polymers one can quote rubber-made car tyres that also contain carbon black and/or silica with the aim of reducing their wear. Also, a new property, foreign to the basic constituents, may be eventually imparted to the resulting material.

Additionally, a new property, foreign to the basic constituents, may be eventually imparted to the resulting material.

Some self-assembling systems are designated as supramolecular polymers [7] since they form thread-like objects (filaments or fibrils) similar to covalent polymers. Mixing these self-assembling systems with covalent polymers appears to be a straightforward way of preparing hybrid materials.

In this paper, we review a new approach consisting of the preparation of hybrid gels in which a thermally reversible polymer gel is the main component wherein the self-assembled system is imbedded. Polymer thermoreversible gels are fibrillar networks with a typical mesh size in the micron range [8]. Their morphology largely resembles organogels, namely an array of fibrils of similar mesh size. In this system, the polymer is used as a matrix, which can lower the cost of the material, enhance the mechanical properties of the hybrid system, and ease tractability [6]. That the hybrid gel contains a large amount of solvent does not stand as a major problem since it can be dried in many ways (simple evaporation, CO_2_ critical extraction…). The solvent can also be recycled as is the case with the making of the PVC fibres (Rhovyl) [9]. Supercritical CO2 extraction can be a good choice as it allows one to keep the original morphology of the hybrid network.

The solvent can also be recycled, as is the case with the making of the PVC fibres (Rhovyl) [9]. Supercritical CO_2_ extraction can be a good choice as it allows one to keep the original morphology of the hybrid network.

**Figure 1:**
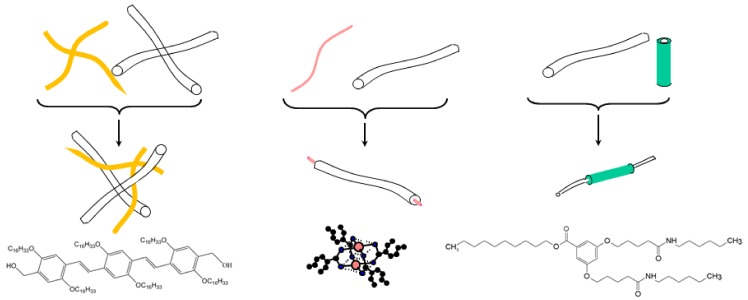
The three types of hybrid materials: left: an intermingled hybrid gel polymer gel with the organogelator shown underneath (designated as OPVOH, yellow); middle: encapsulation of a self-assembled filaments of a bicopper complex molecule shown underneath (pink) designated as CuS8; ● = copper, ● = oxygen; ● = carbon) into a polymer fibril; right: a self-assembled nanotube (green), sheathing a polymer fibril, molecule shown underneath designated as BHPB-10.

In this review, we will present three types of hybrid materials with different ways of using the polymer matrix as portrayed in Figure 1. The first case consisted of preparing an intermingled gel in which the gels pervade one another. In the second case, polymer fibrils encapsulated filaments of a self-assembled bicopper complex. The third case featured the reverse situation, in which self-assembled nanotubes sheathed polymer fibrils. Heterogeneous nucleation was the driving process in the last two cases.

## 2. Preparing Intermingled Gels

**Figure 2:**
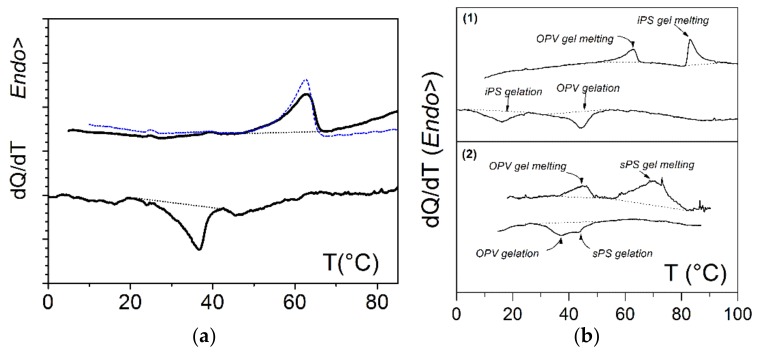
DSC thermograms; (**a**): the OPVOH organogel formation and melting, in blue the gel melting in the ternary system OPVOH/aPS/benzene, where C_aPS_ = 2.5 × 10^−2^ g/cm^3^ and C_OPVOH_ = 0.4 × 10^−2^ g/cm^3^; (**b**) ternary gels: (1) isotactic polystyrene/OPVOH in *trans*-decahydronaphthalene; (2) syndiotactic polystyrene/OPVOH in benzene, where C_polymer_ = 7 × 10^−2^ g/cm^3^ and C_OPVOH_ = 0.4 × 10^−2^ g/cm^3^ (data from Dasgupta et al. [10]). For details, see text.

This section demonstrates and discusses the feasibility of intermingling a polymer thermoreversible gel together with an organogel, the latter bearing an opto-electronic function. The polymer gels are comprised of either isotactic polystyrene (iPS) or syndiotactic polystyrene (sPS). The organogelator is an oligo phenylene vinylene (OPVOH, see Figure 1) which possesses opto-electronic properties, changing colour at the SOL-GEL transition [2]. In the majority of cases, two covalent polymers cannot be mixed on account of entropic grounds. Therefore, the question regarding covalent polymers and supramolecular polymers concerns their degree of compatibility. In other words, can one prepare homogeneous solutions at high temperature that will further gel upon cooling without triggering first a liquid–liquid demixing? A mixture of OPVOH and atactic polystyrene (aPS) was dissolved in benzene by heating the ternary solution (C_OPV_ = 0.004 g/cm^3^, C_aPS_ = 0.04 g/cm^3^) and was subsequently cooled to form the organogel (aPS does not gel under these conditions). As displayed in Figure 2, the formation and melting behaviour of the organogel was not affected by the presence of atactic polystyrene. AFM images also confirm that the gel morphology was not significantly altered by the polymer chains [10].

Therefore, the preparation of polymer/OPVOH intermingled networks was achieved with either types of stereoregular polystyrene (iPS or sPS) considering their different gelation behaviour [8]. They formed in different solvents, and they grew and melted at different temperatures.

**Figure 3:**
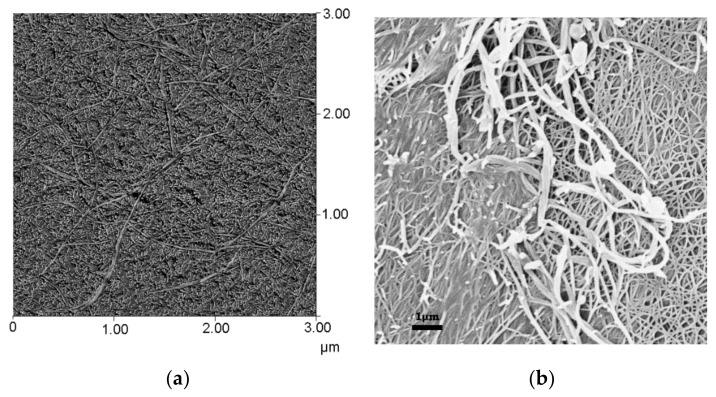
(**a**) AFM picture of an sPS/OPVOH gel in benzene by tapping mode [10]. (**b**) SEM picture of aa sPS/OPVOH gel in p-xylene (courtesy of Christophe Daniel, Universita di Salerno, Italy). In both cases, the fibrils of largest cross-sections were those from the organogel (typically ~200 nm versus ~60 nm).

Typical DSC traces are shown in Figure 2. Again, the presence of a foreign molecule did not perturb the gelation of the other component. The formation and melting temperatures were virtually identical within experimental uncertainties to those observed in binary systems. Further SAXS experiments (not shown) revealed that the scattering by the ternary system was the sum of the scattering by each binary gels. AFM together with SEM investigations presented in Figure 3 [10] clearly demonstrate that the gels pervade one another. Note that the cross-sections of the organogel fibrils were much thicker than those of the polymer gel.

**Figure 4:**
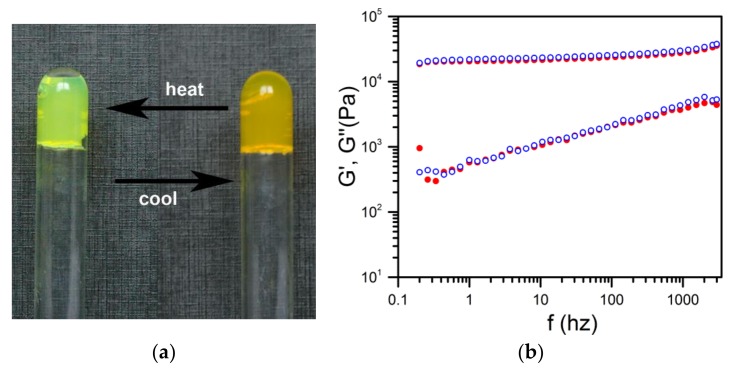
(**a**) Change of colour of the hybrid gel upon heating and cooling cycles. As long as temperature remained below the polymer gel melting point, the hybrid system remained a macroscopic gel [10]. (**b**) Rheological properties of the hybrid gel (◯) versus those of the binary sPS gel (●); the solvent was trans-decahydronaphthalene (unpublished data J.M. Guenet, D. Collin [11], Institut Charles Sadron).

As aforementioned, OPVOH systems change colour at the SOL-GEL transition. This stems from π–π interactions which are established during the gelation process [2]. In the hybrid gel sPS/OPVOH/toluene, the change of colour was again observed, yet without macroscopic melting (Figure 4). DSC data in Figure 2b account for this effect; as long as the temperature was kept below the polymer gel melting point, the OPVOH organogel moiety repeatedly underwent a change of colour at its GEL-SOL-GEL transitions [10].

Rheological experiments carried out by means of a piezzorheometer confirmed that the mechanical properties were essentially the same as those of the polymer gel. The values and the behaviour of both G′ and G′′ of the hybrid gel corresponded within experimental uncertainties to those of the polymer gel [11].

Thus, the proof of concept was demonstrated: intermingled gels from covalent polymer gels and organogels could be prepared thanks to the high degree of compatibility between their components. Accordingly, a functional property was imparted to the polymer matrix with a low amount of organogelator, namely in a *w*/*w* ratio polymer/organogelator of approximately 20.

## 3. Encapsulating Self-Assembled Filaments in Polymer Fibrils

**Figure 5:**
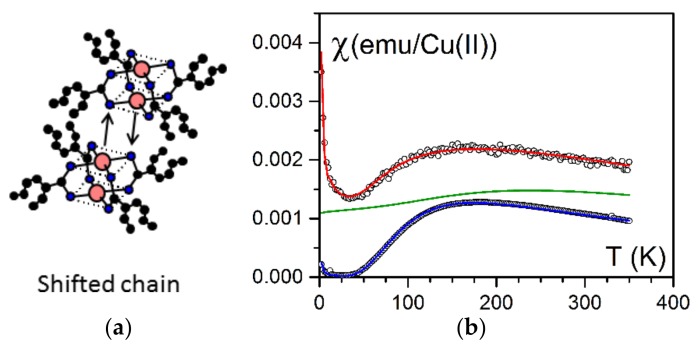
(**a**) How bicopper molecules piled up in the bulk state (shifted chains) [12]. (**b**) Magnetic susceptibility as a function of temperature. Blue = bulk state fitted with a Bleaney-Bowers law; red = encapsulated state fitted by relation (1) (see text) [14,22], green = theoretical variation for a spin chain [20].

The bicopper complex molecule shown in Figure 1 and Figure 5 possesses the propensity of self-assembling in organic solvent, thus generating very long filaments which contain only one molecule in their cross-section [12,13]. The filaments are typical supramolecular polymers; however, as a result of the interaction of the order ok kT between molecules, they are also designated as dynamic polymers since they break and reassemble with the tips of other filaments. The rheological behaviour of their organic solution is essentially that of long covalent polymer chains and is theoretically well reproduced by means of a Maxwell model [13]. Filaments are stable over a long period of time, but 1D filaments gradually disappear to transform into a solid mesophase that eventually phase separates macroscopically.

The bicopper molecule possesses antiferromagnetic properties in the bulk state that are well described by the Bleaney-Bowers equation [14,15]:

(1) $$\chi_{\mathrm{CuS}8}=\frac{N_{A}.g^{2}.\mu_{B}^{2}}{K_{B}T\left[3+\exp\left(\frac{-2J}{K_{B}T}\right)\right]}$$

where N_A_ is Avogadro’s number, g the electron spin factor of the bicopper, J the coupling constant, and µ_B_ Bohr’s magneton.

As relation 1 fitted the experimental results, this indicated that bicopper molecules were magnetically behaving as if they were isolated (spin gap). The small upturn at a very low temperature is generally ascribed to paramagnetic impurities.

Abied et al. have shown that the molecular piling occurs through one copper atom interacting with the oxygen atom of its neighbour [16]. Therefore, no intermolecular Cu–Cu interactions were involved in the bulk state.

Although the one-dimensional aspect of the filaments might be of interest for testing their magnetic properties, their instability over time, together with the impossibility of keeping them apart after solvent removal, made this a remote option. The possibility of encapsulating these filaments into polymer fibrils was contemplated by Lopez and Guenet thanks to existence of a common solvent a solvent, decahydronaphthalene, where the bicopper complex piles up into 1-D filaments while isotactic polystyrene forms a thermoreversible gel. [17]. It has been shown that the bicopper complex and the polymer form homogeneous solutions at high temperatures and are largely compatible at lower temperatures [18,19].

The encapsulation of bicopper filaments into the polymer fibrils uses a heterogeneous nucleation process. The first filaments to form have been shown to act as an impurity towards the polymer and accordingly trigger the growth of the fibrils. Otherwise, polymer gel formation is driven by a homogeneous nucleation process. A heterogeneous nucleation mechanism implies that the impurity, namely the filaments, are located in the central core of the fibrils. DSC experiments have confirmed the occurrence of heterogeneous nucleation [17,20] by revealing the increase of the gel formation temperature while increasing the bicopper complex content.

**Figure 6:**
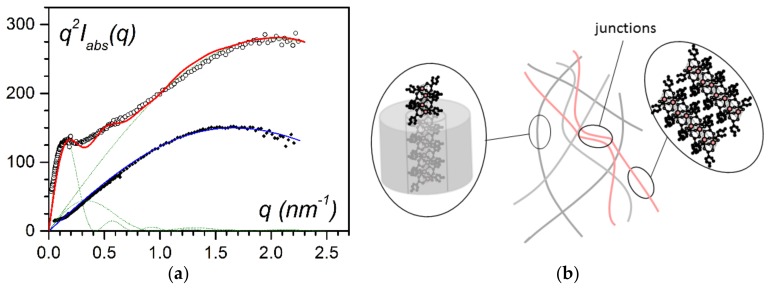
(**a**) Small-angle neutron scattering data for IPS/CuS8/trans-decahydronaphthalene gels: in all cases C_iPS_ = 0.04 g/cm^3^, ● for C_CuS8_ = 0.038 g/cm^3^, ◯ for C_CuS8_ = 0.055 g/cm^3^. The solid lines (red and blue) fit with theoretical models (see text for details) [17,20]. (**b**) Sketch of the molecular structures for C_CuS8_ = 0.055 g/cm^3^: encapsulated fibrils, free-associated filaments and their junctions. The encapsulated fibrils represent 99% of the structures.

Small-angle neutron-scattering experiments have allowed the determination of the structure of both the polymer and bicopper complex by a proper matching contrast approach. These investigations show that the average polymer fibril cross-section decreases proportional to an increase in bicopper content [21], an outcome which agrees with a heterogeneous nucleation process. It also suggests that the central part of the fibrils consists of a different constituent, namely the bicopper complex. They further show that the filament structure is preserved (Figure 6) as, for CuS8 concentrations below C_CuS8_ = 0.04 g/cm^3^, the scattering curve can be fitted with a simple solid cylinder model:

(2) $$q^{2}I_{\mathrm{abs}}\left(q\right)=4{\pi \mathrm{qC}}_{\mathrm{CuS}8}\mu_{\mathrm{fil}}\times \frac{J_{1}^{2}\left({\mathrm{qr}}_{\mathrm{fil}}\right)}{q^{2}r_{\mathrm{fil}}^{2}}$$

where µ_fil_ and r_fil_ are the mass per unit length and the cross-sectional radius of the filaments, respectively, and J_1_ the Bessel of first kind and first order.

The fit of the experimental data yields µ_fil_ = 1300 ± 100 g·mol^−1^·nm^−1^ and r_fil_ = 0.7 ± 0.1 nm, which confirms the near one-dimensional structure of the encapsulated filaments [20].

By increasing the bicopper complex content, a portion of the filaments was no longer encapsulated. Figure 6b shows the additional scattering due to the presence of associated one-dimensional filaments and to their sporadic junctions. Accordingly, the scattering curve can be fitted with the corresponding three types of structures:

(3) $$q^{2}I_{\mathrm{abs}}\left(q\right)=4{\pi \mathrm{qC}}_{\mathrm{CuS}8}\left[{X\mu }_{\mathrm{fil}}\times \frac{J_{1}^{2}\left({\mathrm{qr}}_{\mathrm{fil}}\right)}{q^{2}r_{\mathrm{fil}}^{2}}+{Y\mu }_{\mathrm{fib}}\times \frac{J_{1}^{2}\left({\mathrm{qr}}_{\mathrm{fib}}\right)}{q^{2}r_{\mathrm{fib}}^{2}}+{Z\mu }_{\mathrm{junc}}\times \frac{J_{1}^{2}\left({\mathrm{qr}}_{\mathrm{junc}}\right)}{q^{2}r_{\mathrm{junc}}^{2}}\right]$$

where µ and r stand with appropriate subscripts for the mass per unit length and for the cross-sectional radius of the encapsulated filaments, the associated filaments, and the junctions. X, Y, Z are the fractions of each species.

The fit yields r_fil_ = 0.67 nm, r_fil_ = 4 nm, and r_junc_ = 10 nm, μ_fil_ = 1300 g/mol·nm,·μ_fib_ = 7500 g/mol·nm and μ_junc_ = 46800 g/mol·nm, and X = 0.95, Y = 0.03, and Z = 0.02 [20].

**Figure 7:**
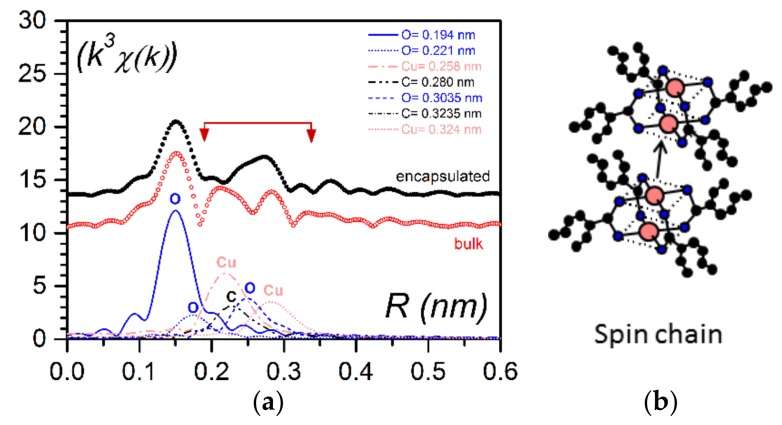
(**a**) Pseudo-radial distribution functioned in the vicinity of copper atoms as obtained by EXAFS; ● for the encapsulated bicopper complex, ◯ for the bulk state (note the systematic difference of approximately 0.05 nm in EXAFS with the actual distances). The difference between the curves occurred in the domain involving Cu–Cu distances (**b**) a spin chain where the Cu–Cu interaction between adjacent molecules occurred in lieu of the Cu–O interaction seen in the bulk state [20,22].

As shown in Figure 5, the magneric behaviour differed drastically whether the bicopper complex was in the bulk state or was encapsulated. It was suspected that the piling of the molecules in the encapsulated filaments may have differed from that in the bulk state [22]. Boulaoued et al. investigated this issue [20] and performed EXAFS (Extended X-ray Absorption Fine Structure [23]) experiments for determining the environment of the copper atoms. While the model put forward by Abied et al. for the bulk state was confirmed [16], the experimental results conspicuously differed in the encapsulated state. It was concluded that Cu–O interactions between adjecent molecules were replaced to a certain extent by Cu–Cu interactions (Figure 7a). Thus, the encapsulated filaments were no longer purely shifted chains (Figure 5a) but contained spin chains (Figure 7b).

From these conclusions, Boulaoued et al. could fit their SQUID data by using a model where three species were analyzed: (i) bicopper molecules with only intramolecular coupling (spin gap due to shifted chains) with 2J_intra_ the coupling constant. (ii) Antiferromagnetic spin chains with 2J_intra_ and 2J_inter_, namely inter and intra coupling constants. The alternation parameter α = 2J_inter_/2J_intra_ was equal to one in the case of a spin chain (gapless spin) and amounted to α = 0 for non-coupled dimers (shifted chains, spin gapped); (iii) paramagnetic impurities. The magnetic susceptibility is then written:

*χ_hybrid_ = xχ_spingap_ + yχ_gapless_ + zχ_pm_* (4)

with obvious meaning as to the subscripts (pm= paramagnetic impurities), *x*, *y*, *z*, being the fraction of each species (further details in supp. info. of ref. [20]). The fit yields 2J = −196 ± 5 cm^−1^ for the shifted chains and 2J_intra_ = 2J_inter_ = −205 ± 5 cm^−1^ for the spin chain, with *x* = 0.54, *y* = 0.45, *z* = 0.01. The values of the coupling constant are consistent with the value found in the bulk state (2J = −198 ± 5 cm^−1^). The value of g was kept at *g* = 2.17 as determined independently by ESR.

Therefore, the fit indicates that about half of the filaments, or parts of the filaments, piled in a different fashion. The origin of this effect in the encapsulated state is still unknown. It is suspected that the chains surrounding the filaments exerted some constraint, which eventually modified the original piling [20]. It is worth emphasizing that such a magnetic effect is usually found in highly organized systems (crystals). Its observation in a gel is probably the first occurrence observed in a randomly dispersed system.

## 4. Sheathing Polymer Fibrils with Self-Assembled Systems

### 4.1. Case of Isotactic Polystyrene: A Neutral Polymer

**Figure 8:**
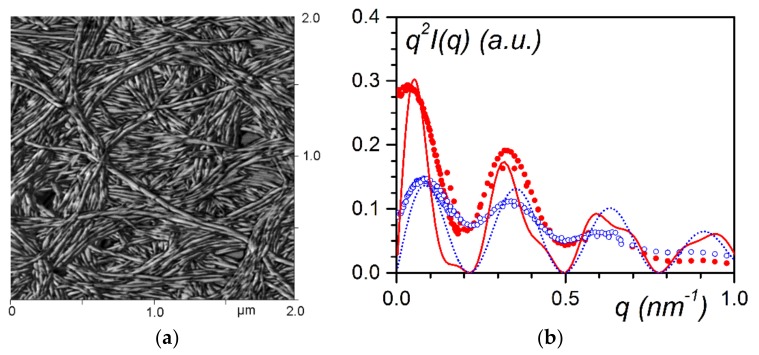
(**a**) AFM picture of nanotubes formed in trans-decahydronaphthalene (Guenet unpublished data); (**b**) Scattering curves plotted by means of a Kratky representation q^2^I(q) vs. q [25]; (●) phbp-10/trans-decahydronaphthalene_D_ system, (◯) iPS_D_/bhpb-10/trans-decahydronaphthalene_D_ hybrid gel. Blue dotted line = fit with Equation (5), red solid line = fit with Equation (6).

Recently, Mésini and coworkers have synthesised molecules (bhpb-10 see Figure 1) capable of forming nanotubes with very narrow inner and outer radii distributions [24] (Figure 8a). The occurrence of these nanotubes is clearly demonstrated by small-angle scattering experiments (Figure 8b).

The scattering curve of the bhpb-10/trans-decahydronaphthalene_D_ system can be fitted with a bunch of parallel hollow cylinders [25]:

(5) $$q^{2}I\left(q\right)=\left[2{\pi \mathrm{qC}\mu }_{L}{\left[\frac{2}{\left(1-\gamma^{2}\right)r_{\mathrm{ext}}}\times \left\{J_{1}\left({\mathrm{qr}}_{\mathrm{ext}}\right)-{\gamma J}_{1}\left({q\gamma r}_{\mathrm{ext}}\right)\right\}\right]}^{2}\right]\times \overset{n}{\underset{j=1}{\sum }}\sum_{k=1}^{n}J_{o}\left({\mathrm{qr}}_{\mathrm{jk}}\right)$$

The bracketed first term stands for the scattering by a hollow cylinder where r_out_ is the external radius, γr_out_ the value of the inner radius, C the concentration, and µ_L_ the mass per unit length. J is the Bessel function of the first kind, with J_1_ of first order, and J_o_ of zeroth order. The second term in relation (5) represents intermolecular interactions between pairs of parallel cylinders. Indeed, as can be seen in Figure 8a, several nanotubes are parallel to one another, a fact which must be taken into account.

**Figure 9:**
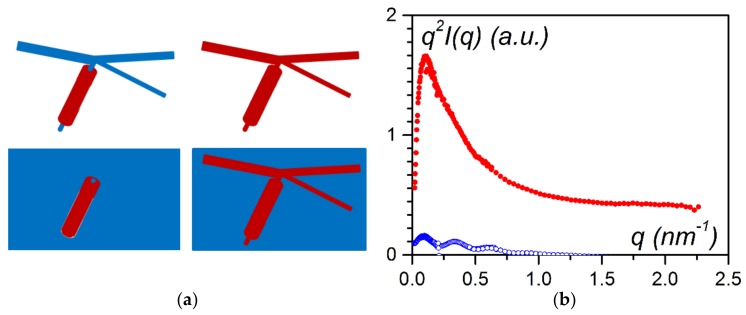
(**a**) Sketch of nanotubes sheathing polymer fibrils. The nanotubes are randomly oriented. The colours indicate hydrogenous species (red) and deuterated species (blue) to illustrate the contrast matching. In a deuterated solvent (blue), only the hydrogeneous species are seen. (**b**) Scattering curves for iPS_D_/bhpb-10/trans-decahydronaphthalene_D_ (◯) and iPS_H_/bhpb-10/trans-decahydronaphthalene_D_ (●) plotted by means of a Kratky representation q^2^I(q) vs. q [25].

DSC experiments carried out on the hybrid system have suggested the occurrence of a heterogeneous nucleation effect, which is consistent with a sheathing process [25]. AFM investigations have shown an increase of the polymer fibrils cross-sections. However, this is only circumstantial evidence. Neutron-scattering experiments performed by varying the contrast of the different species have allowed the confirmation of the sheathing of the polymer fibrils. By using an isotopic solvent mixture that matches the coherent signal of either species (Figure 9a), Dasgupta et al. have derived the following conclusions [25]:

(i) Matching the coherent scattering of deuterated isotactic polystyrene (iPS_D_) with deuterated trans-decahydronaphthalene_D_ allows one to determine the structure of bhpb-10 molecules in the ternary system. The scattering curve differs from that recorded in the binary system (Figure 8b) as it can still be fitted with a hollow cylinder model, yet without considering intermolecular terms. This indicates that parallel cylinders are absent, as would be the case if the nanotubes were randomly oriented through the sheathing process (see Figure 9a).

(ii) When hydrogeneous iPS is used instead of deuterated iPS, the oscillations previously observed disappear (Figure 9b). Here iPS and bhpb-10 have virtually the same scattering amplitude so that they can no longer be distinguished. There is an absence of oscillations which is explained in Figure 9a. As a result of their well-defined inner diameter, bhpb-10 nanotubes can only sheathe those fibrils with adequate cross-sections. As there is a large cross-section dispersity [21], oscillations are dampened and eventually completely vanish. If the nanotubes were independent of the polymer structure, oscillations would be superimposed onto the iPS scattering.

The neutron-scattering experiments, together with the contrast matching, provide direct evidence that nanotubes do sheathe a fraction of the polymer fibrils. The mechanism involved in the sheathing process is likely the hetergeneous nucleation of the nanotubes by the polymer fibrils.

### 4.2. Case of P3BT (Poly[3-butylthiophene-2,5-diyl]): A Semi-Conducting Polymer

The case of iPS fibrils sheathed by nanotubes has been useful for establishing a proof of concept, but applications are not in view for such a system. Conversely, sheathing semi-conducting polymers, which allow for the preparation of semi-conducting nanowires, seems a more promising perspective in the field of functional materials. Exploring new paths in the processing of these polymers may offer new opportunities for application purposes.

**Figure 10:**
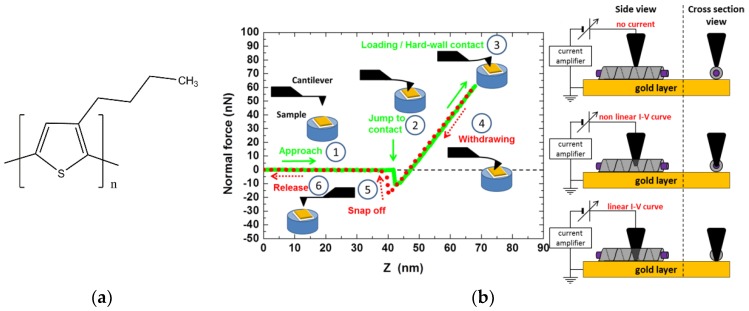
(**a**) Chemical structure of P3BT. (**b**) Right: the tip of the C-AFM is gradually allowed to penetrate into the selected fibril until it reaches the gold layer. The current and the force are simultaneously measured to determine the conductivity and the penetration depth, respectively (for details see reference [26]). Copyright 2018 John Wiley & Sons.

Raj, Boulaoued, and coworkers [26] studied the possibility of sheathing fibrils of P3BT (Poly[3-butylthiophene-2,5-diyl] Figure 10a) which is well-known in organic electronics as a p-type material [27].

As the P3BT fibril grew somewhat slowly, the preparation procedure of the hybrid system involved three steps: first, the P3BT fibres were allowed to grow for several hours at room temperature; second, the system was heated to 60 °C at which point a bhpb-10 solution was added at the same temperature (no nanotubes were formed at this temperature); third, the mixture was cooled to room temperature to trigger the formation of nanotubes.

Two different types of investigations were subsequently performed: conducting AFM (C-AFM) on diluted solutions and SANS on more concentrated solutions.

**Figure 11:**
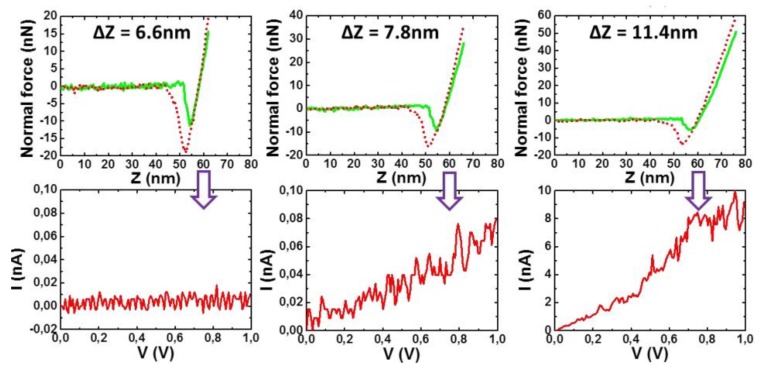
I-V plots (lower curves) vs. penetration depth (upper curves). From left to right: insulator behaviour, appearance of current, semi-conductor behaviour [26]. Copyright 2018 John Wiley & Sons.

The principle of C-AFM is shown in Figure 10b. Once a supposedly sheathed fibril was spotted, the tip of the C-AFM was allowed to penetrate it gradually while the current was measured (intensity vs. voltage, I-V curve). The distance Z after the “jump to contact” was the sum of cantilever deflection and of the tip penetration/indentation depth (see Figure 10b). Figure 11 shows that the tip first encountered an isolating layer until it reached a certain depth where the I-V curve corresponded to a semi-conducting system. This result provided a strong indication that P3BT fibrils were sheathed by bhpb-10 nanotubes.

**Figure 12:**
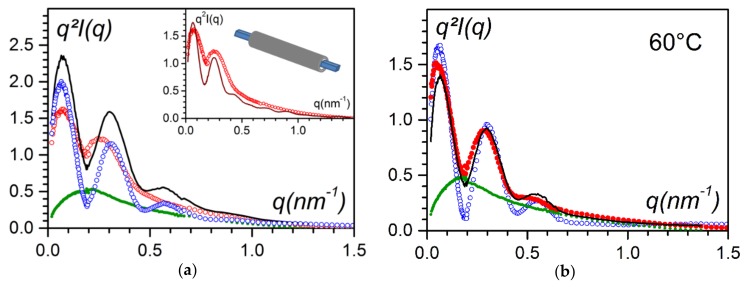
SANS curves plotted by means of a Kratky-plot, q^2^I(q) vs q. For both figures: ◯ = bhpb-10/trans-decahydronaphthalene; ◯ = hybrid system; ● = P3BT fibrils in trans-decahydronaphthalene; solid black line = sum of the scattering curves of the binary systems (● + ◯). (**a**) Results obtained at T = 20 °C, inset fit of the scattering curve of the hybrid system with the model shown (blue = P3BT fibrils, grey = nanotube; see text for details). (**b**) Results at 60 °C [26].

An additional experiment was carried out by mapping the current that flowed across grounded and isolated gold electrodes, separated by an insulating 5 µm trough [26]. Through the use of contact mode C-AFM, current maps were concomitantly recorded with the topography. When pure bhpb-10 was deposited between the electrodes, no current was detected in the trough nor in the isolated electrode. When P3BT fibrils were deposited, current was detected in the trough and in the isolated electrode. When the hybrid system was deposited, no current was detected in the trough while current was measured on the isolated electrode. This gives a strong indication of the existence of nanotube-sheathed P3BT fibrils that can be described as insulated molecular wires (see inset of Figure 12a).

SANS experiments also confirmed the sheathing process. P3BT are known to form fibrils of rectangular cross-sections [28,29], which was confirmed by a fit of the scattering curve with [30]:

(6) $$q^{2}I\left(q\right)\sim \;\frac{2{q\text{µ}}_{L}}{\pi }\int_{o}^{\pi /2}{\left[\frac{\sin\mathrm{qa}/2\cos\theta }{\mathrm{qa}/2\cos\theta }\times \frac{\sin\mathrm{qb}/2\sin\theta }{\mathrm{qb}/2\sin\theta }\right]}^{2}\sin\theta d\theta$$

which yielded a = 4 nm and b = 14 nm, corresponding to a diagonal of about 15 nm.

Figure 12a shows that the scattering curve of the hybrid system differed from the simple sum of the scattering curves of the P3BT fibrils and of the bhpb-10. Clearly, the ternary system was not a mixture of P3BT fibrils and bhpb-10 nanotubes.

Conversely, at 60 °C, the scattered intensity of the ternary system was the sum of the scattered intensity of each component in the binary systems scaled by a factor of 0.7. This indicates that the hybrid entity observed at the lower temperature represented about 30% of the components.

In the inset of Figure 12a a tentative fit is perfomed considering nanotubes sheathing the P3BT fibrils by using the following relation:

(7) $$I_{\mathrm{hybrid}}^{\mathrm{theo}}\left(q\right)\sim \frac{{\pi \mu }_{\mathrm{LHyb}}}{q}{\left[\frac{2{\gamma A}_{P3\mathrm{BT}}}{A_{m}{\mathrm{qr}}_{\mathrm{out}}}J_{1}\left({q\gamma r}_{\mathrm{out}}\right)+\frac{2A_{\mathrm{bhpb}-10}}{A_{m}{\mathrm{qr}}_{\mathrm{out}}}\times \left(J_{1}\left({\mathrm{qr}}_{\mathrm{out}}\right)-{\;\gamma J}_{1}({\gamma \mathrm{qr}}_{\mathrm{out}}\right)\right]}^{2}$$

where A_P3BT_ and A_bhpb-10_ are the scattering amplitude of the P3BT moiety and of the bhpb-10 molecules, with $A_{m}=\gamma^{2}A_{P3BT}+\left(1-\gamma^{2}\right)A_{bhpb-10}$, r_out_ the outer radius of the hybrid system (namely, the sheathing nanotubes), and γr_out_ the inner radius of the sheathing nanotube. Considering this only represents 30% of the hybrid system, the intensity should be written:

(8) $$I\left(q\right)=0.35\times I_{P3BT}^{exp}\left(q\right)+0.35\times I_{BHPB-10}^{exp}\left(q\right)+0.3\times I_{hybrid}^{theo}\left(q\right)$$

The foremost goal in the fitting procedure was to match the positions of the first two maxima; under these conditions, one obtains r_out_ = 20 nm and γr_out_ = 9 nm with a relatively good agreement with the magnitude of the intensity. The inner radius was consistent with the half-value of the diagonal of the P3BT ribbons (7.5 nm). The outer radius is about 1.6 larger than the outer radius of the pure nanotubes, which gives a layer of about 11 nm, a value in agreement with the outcomes of the C-AFM data.

The C-AFM outcomes suggest that most of the P3BT fibrils were sheathed, while only 30% of hybrid material needed to be considered to account for neutron-scattering results. This might be because the concentration domains differed by about two orders of magnitude. Additionally, the hybrid material disappeared at 60 °C while P3BT fibrils and bhpb-10 nanotubes were still present. That the hybrid system melted at lower temperature is reminiscent of the behaviour of a eutectic compound. This further backs up the occurrence of the sheathing process as an eutectic compound, requires an intimate mixture of two components. 

### 4.3. Case of Syndiotactic Polystyrene with A Partially-Fluorinated Self-Assembled System

**Figure 13:**
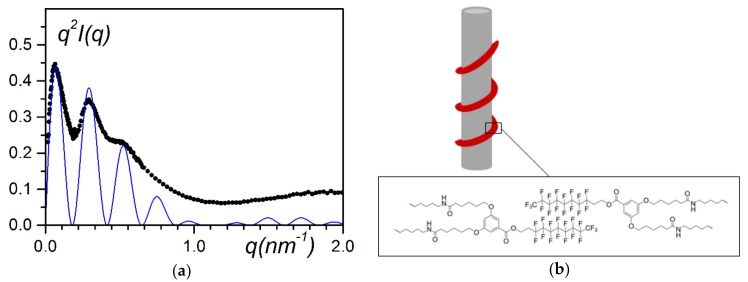
(**a**) SANS data plotted by means of a Kratky-plot, q^2^I(q) vs. q.; ● = sPSD/bhpbfH/o-xyleneD (C_bhpbfH_ = 0.01 g/cm^3^, C_sPSD_ = 0.15 g/cm^3^); solid line= fit with Equation (9). (**b**) Model designated as “bindweed”, where bhpbf irregular helices wind around polymer fibrils [31].

Another system involving a partially fluorinated bhpb-10 (designated as bhpbf, see Figure 13b) and syndiotactic polystyrene (sPS) was investigated by Khan et al. with the aim of preparing highly hydrophobic materials. While the expected target was not reached, nanohybrid materials were still obtained [31]. Unlike bhpb-10, bhpbf does not form nanotubes but twisted helices, where the fluorinated moiety is located in the core [32].

Among the various types of experiments performed to determine the structure of the hybrid system, several results from the neutron-scattering data are worth mentioning. The system was prepared from deuterated syntiotactic polystyrene (sPS_D_), hydrogeneous bhpbf, and deuterated p-xylene, so that the main contribution arises from the bhpbf moiety. Khan et al. have shown that the intensity can be fitted with the equation of a hollow cylinder [32]:

(9) $$q^{2}I\left(q\right)=2{\pi \mathrm{qC}\mu }_{L}{\left[\frac{2}{\left(1-\gamma^{2}\right)r_{\mathrm{ext}}}\times \left\{J_{1}\left({\mathrm{qr}}_{\mathrm{ext}}\right)-{\gamma J}_{1}\left({q\gamma r}_{\mathrm{ext}}\right)\right\}\right]}^{2}$$

where r_ext_ is the external radius and all other parameters are equivalent to previous equations.

This points towards an irregular helical structure winding around the polymer fibrils (the bindweed model), as the derived mass per unit length, µ_L_ ≈ 2 × 10^3^ g/mol·nm, is not consistent with a nanotube for which one would obtain µ_L_ ≈ 3.4× 10^5^ g/mol·nm. Again, because of the low resolution in the explored q-range, a helical structure, regular or irregular, scattered like a hollow cylinder (it corresponded to the zeroth order layer line). Additionally, the fitting procedure consisted of matching above all the position of the maxima of the oscillations, which yielded r_ext_ = 16 nm with γ = 0.65. Notably, the fit allows close reproduction of the scattering envelope. The fit could be improved by considering radius dispersity. The thickness δ of the helical structure, namely δ = r_ext_ − γr_ext_ = 5.6 nm, was in agreement with the helical parameters of bhpbf reported earlier [32], which lends additional support to the validity of the fitting procedure.

Note that the polymer fibrils were essentially sheathed in a similar manner by the self assembling molecules, whether these were intrinsically forming nanotubes (bhpb-10) or not (bhpbf). To some extent, the irregular helix of bhpbf could be regarded as an open nanotube.

## 5. Conclusions

This short review highlights the possibility of preparing a large variety of hybrid systems from covalent polymers as well as self-assembling molecules through the simple implementation of physical processes such as heterogeneous nucleation and thermoreversible gelation. This preparation method could be achieved thanks to the high compatibility of the components in the SOL state. By virtue of intrinsic gel morphology, which consists of an array of randomly dispersed thin fibrils, the resulting molecular structures typically fell within the nanometre range. Functional materials could thus be obtained, where the functionality was provided by the self-assembled moiety and most notably at a relatively low concentration. Thanks to the nanometre aspect, a new, unexpected magnetic property was imparted to the bicopper complex in the hybrid system. To date, such a magnetic property had been only observed in highly organized systems. In the case of P3BT, insulated semi-conducting nanowires could be prepared solely through a physical process, which provides a novel, hitherto unexplored way of processing this polymer.

The examples presented herein may pave the way for preparing functional materials in a rather easy way without resorting to complex chemical synthesis.
